# PHYSICAL ACTIVITY AND DEMENTIA IN THE NATIONAL HEALTH AND AGING TRENDS STUDY (NHATS)

**DOI:** 10.1093/geroni/igad104.1171

**Published:** 2023-12-21

**Authors:** Anis Davoudi, Patrick Donahue, Michelle Carlson, Ryan Dougherty, Amal Wanigatunga, Vicki Freedman, Jennifer Schrack

**Affiliations:** Johns Hopkins University, Baltimore, Maryland, United States; Johns Hopkins University, Baltimore, Maryland, United States; Johns Hopkins Bloomberg School of Public Health, Baltimore, Maryland, United States; Johns Hopkins University, Baltimore, Maryland, United States; Johns Hopkins School of Public Health, Baltimore, Maryland, United States; University of Michigan, Ann Arbor, Michigan, United States; Johns Hopkins University, Baltimore, Maryland, United States

## Abstract

Individuals living with dementia exhibit disrupted sleep/wake rhythms; however, whether daily physical activity volume and patterns are worse among those living with dementia is not well established. We examined the cross-sectional association between dementia classification and accelerometry-derived patterns of daily physical activity in NHATS. Wrist-worn accelerometry was collected using the Actigraph Centrepoint Insight Watch in a subset of Round 11 (2021) participants. Dementia status, including possible and probable dementia, was determined using the NHATS dementia algorithm. We characterized daily physical activity patterns using: most active 10-hours/day (M10), time of M10, least active 5-hours/day (L5), time of L5, relative amplitude (RA), intra-daily variability (IV), inter-daily stability (IS), and activity fragmentation using the active-to-sedentary transition probability (ASTP). We used separate linear regression models to study the association between dementia and each actigraphy feature, adjusting for relevant demographic features including age, gender, race, BMI, and education. 721 participants had ≥3 days of valid accelerometry data, dementia status, and complete covariate data (40% aged ≥80 years, 54% women). Compared to cognitively normal participants, those living with dementia averaged fewer activity counts/day (beta: -382K counts/day p<0.0001), lower M10 (beta: -261K counts, p<0.0001), lower IS (beta: -0.04; p= 0.0066), and higher ASTP (beta: 7.1%; p<0.0001). Participants with and without dementia did not differ in terms of IV, L5, or RA (p>0.05 for all). These results suggest important differences in volume and patterns of daily movement by dementia status. Future studies should investigate whether similar differences exist in earlier stages of cognitive impairment to enhance preventative efforts.

